# Biochemical Characterization and Degradation Pattern of a Unique pH-Stable PolyM-Specific Alginate Lyase from Newly Isolated *Serratia marcescens* NJ-07

**DOI:** 10.3390/md16040129

**Published:** 2018-04-15

**Authors:** Benwei Zhu, Fu Hu, Heng Yuan, Yun Sun, Zhong Yao

**Affiliations:** College of Food Science and Light Industry, Nanjing Tech University, Nanjing 211816, China; hufu@njtech.edu.cn (F.H.); yuanheng17@njtech.edu.cn (H.Y.); sunyun_food@njtech.edu.cn (Y.S.)

**Keywords:** *Serratia marcescens*, polyM-specific, alginate lyase, oligosaccharides

## Abstract

Enzymatic preparation of alginate oligosaccharides with versatile bioactivities by alginate lyases has attracted increasing attention due to its featured characteristics, such as wild condition and specific products. In this study, AlgNJ-07, a novel polyM-specific alginate lyase with high specific activity and pH stability, has been purified from the newly isolated marine bacterium *Serratia marcescens* NJ-07. It has a molecular weight of approximately 25 kDa and exhibits the maximal activity of 2742.5 U/mg towards sodium alginate under 40 °C at pH 9.0. Additionally, AlgNJ-07 could retain more than 95% of its activity at pH range of 8.0–10.0, indicating it possesses excellent pH-stability. Moreover, it shows high activity and affinity towards polyM block and no activity to polyG block, which suggests that it is a strict polyM-specific alginate lyase. The degradation pattern of AlgNJ-07 has also been explored. The activity of AlgNJ-07 could be activated by NaCl with a low concentration (100–300 mM). It can be observed that AlgNJ-07 can recognize the trisaccharide as the minimal substrate and hydrolyze the trisaccharide into monosaccharide and disaccharide. The TLC and ESI-MS analysis indicate that it can hydrolyze substrates in a unique endolytic manner, producing not only oligosaccharides with Dp of 2–5 but also a large fraction of monosaccharide. Therefore, it may be a potent tool to produce alginate oligosaccharides with lower Dps (degree of polymerization).

## 1. Introduction

Alginate is the major component of cell wall of brown algae [1]. It is a linear anionic polysaccharide and consists of α-l-guluronate (G) and its C5 epimer β-d-mannuronate (M), which are linked by α-1, 4-glycosidic bonds [2]. The two monomeric units are arranged into three groups: poly-α-l-guluronate (polyG), poly-β-d-mannuronate (polyM), and the heteropolymer (polyMG) [3]. Due to its high viscosity, gelling properties, and versatile activities, alginate has been widely applied in food, chemical, and pharmaceutical industries [4,5,6]. However, the applications of this polysaccharide are still limited by its high molecular weight and poor solubility [7]. The alginate oligosaccharide, as the degradation product of alginate, retains various specific physiological functions and activities of polysaccharide but possesses good bioavailability [8]. For instance, Pack et al. found that alginate oligosaccharide (AOS) can reduce plasma LDL-cholesterol levels by regulating the expression of LDLR [9]. Iwamoto et al. studied the effect of AOS with different structures on the induction of cytokine production from RAW264.7 cells and found that G8 and M7 showed the most potent activity [10]. Yamamoto et al. reported that mannuronate oligomers (M3–M7) could induce the production and secretion of multiple cytokines, such as tumor necrosis factor- α (TNF-α), granulocyte colony-stimulating factor (GCSF), and monocyte chemoattractant protein-1 (MCP-1) [11].

Alginate lyase, a member of polysaccharide lyase, can catalyze the alginate by the β-elimination, producing unsaturated oligosaccharides with double bonds between C4 and C5 [12]. Until now, a number of alginate lyases have been identified, gene-cloned, purified, and characterized from various sources, such as marine and terrestrial bacteria, marine mollusks, and algae [13,14,15,16,17,18]. According to the substrate specificities, alginate lyases can be classified into three types: polyM-specific lyases (EC 4.2.2.3), polyG-specific lyases (EC 4.2.2.11), and bifunctional lyases (EC 4.2.2.-) [19]. Additionally, the alginate lyases are generally organized into seven polysaccharide lyase (PL) families according to the sequence similarity, namely PL-5, -6, -7, -14, -15, -17, and -18 families [20]. Moreover, in terms of the mode of action, alginate lyases can be grouped into endolytic and exolytic alginate lyases [21]. Endolytic enzymes can cleave glycosidic bonds inside alginate polymer and release unsaturated oligosaccharides as main products [22], while exolytic ones can further degrade oligosaccharides into monomers [23]. Now alginate lyases, especially endolytic enzymes, have been widely used to produce alginate oligosaccharides for food and nutraceutical industries [24,25]. Moreover, the enzymes can also be used to elucidate the fine structures of alginate and prepare protoplast of brown algae [26,27,28]. Furthermore, alginate lyases also show great potential in the treatment of cystic fibrosis by degrading the polysaccharide biofilm of pathogen bacterium [29]. So far, many alginate lyases originating from marine microorganisms have been well characterized. However, few of these enzymes have been commercially used in the food and nutraceutical industries due to the poor substrate specificity and low activity [30,31,32,33,34,35,36]. Thus, to explore novel enzymes with high activity and high substrate specificity will be of great importance for both research and commercial purposes.

In this work, a new alginate lyase with high substrate specificity and pH stability has been identified and characterized from *Serratia marcescens* NJ-07. To evaluate the enzyme for potential use in the food and nutraceutical industries, the kinetics and analysis of degrading products has also been characterized, which suggests that it would be a potential candidate for expanding applications of alginate lyases.

## 2. Results and Discussions

### 2.1. Screening and Identification of Strain NJ-07

**Figure 1:**
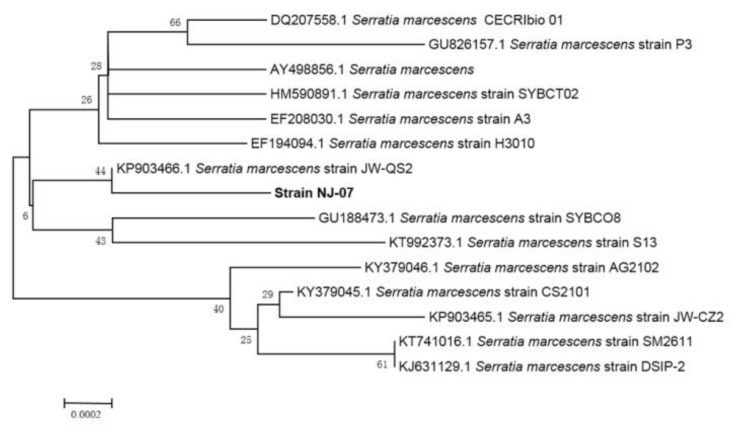
The phylogenetic analysis of strain NJ-07 and other similar strains. The phylogenetic tree was constructed by MEGA 6.0 on the basis of the 16S rRNA gene sequences of strain AlgNJ-07 and other known Serratia species.

The strain was isolated from rotten red algae from the Yellow Sea. The 16S rRNA sequence of the strain was sequenced and submitted to GeneBank (accession number MH119760). According to the phylogenetic analysis of 16S rRNA sequence (Figure 1), the strain was assigned to the genus Serratia and named *Serratia marcescens* NJ-07.

### 2.2. Purification of Alginate Lyase

**Figure 2:**
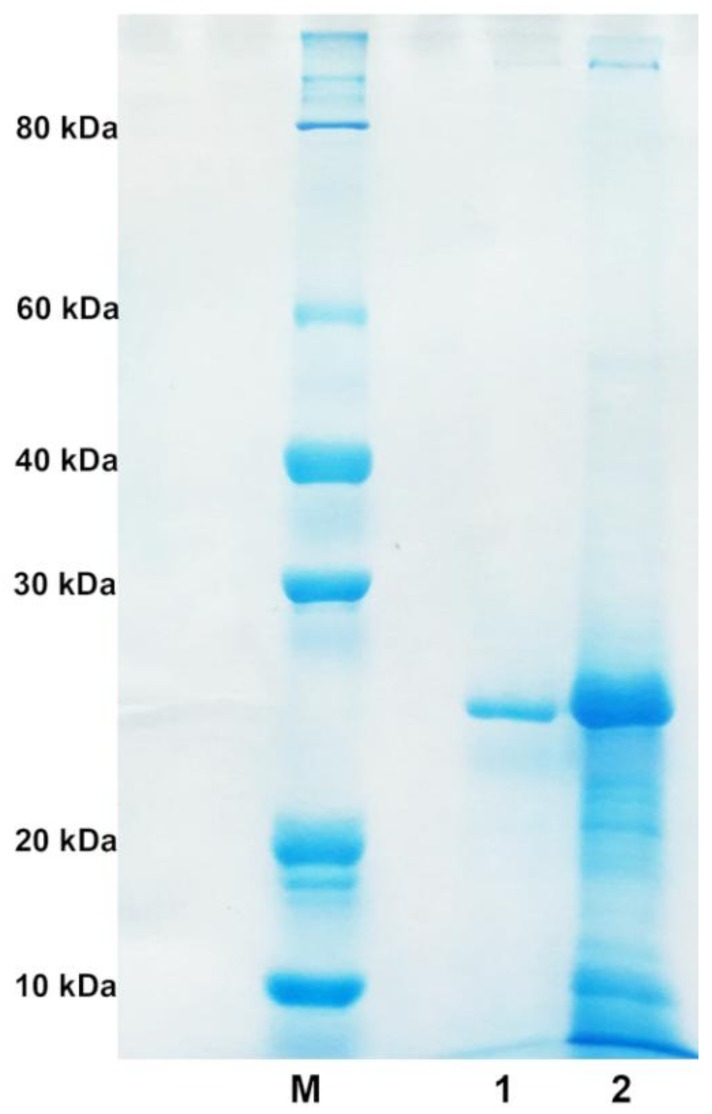
The SDS-PAGE analysis of purified alginate lyase AlgNJ-07. Lane M: the protein molecular weight standard; lane 1: the purified AlgNJ-07; lane 2: the crude enzyme from supernatant.

The strain NJ-07 was cultured in optimized liquid medium for 40 h until alginate lyase reached the highest activity. The supernatant containing alginate lyase was subjected to further purification by anion exchange chromatography with Source 15Q. After purification, the alginate lyase was purified 7.43-fold with a yield of 68.1%. The final specific activity of the purified alginate lyase was 2742.5 U/mg towards sodium alginate. The result of SDS-PAGE showed a single protein band with a molecular weight of 25 kDa (Figure 2), which was designated as AlgNJ-07. The alginate lyases are grouped into three types based on their molecular weights: small alginate lyases (25–30 kDa), medium-sized alginate lyases (around 40 kDa), and large alginate lyases (>60 kDa). As a result, the AlgNJ-07 belongs to the small ones. Similarly, the AlyA from *Azotobacter chroococcum* 4A1M has a small molecular weight of 24 kDa [31]. While the AlyA from *Pseudomonas* sp. E03, AlyA from *Pseudomonas aeruginosa*, and AlyA from *Pseudomonas* sp. strain KS-408 possess the medium-sized molecular weights of 40.4 kDa, 43 kDa, and 44.5 kDa, respectively [33,34,36]. The ALYII from *Pseudomonas* sp. OS-ALG-9 has a large molecular weight of 79 kDa [32].

### 2.3. Substrate Specifity and Enzymatic Kinetics of the Enzyme

Seven kinds of polysaccharide substrates were used to investigate the substrate specificity of the enzyme (Table 1). The alginate lyase showed higher activity towards sodium alginate and polyM, but no activity towards polyG. Additionally, the AlgNJ-07 displayed no activity towards pullulan, pectin, xylan, and heparin. Therefore, the AlgNJ-07 is a novel polyM-specific alginate lyase. Until now, hundreds of alginate lyases have been identified and characterized. However, only a few enzymes exhibited the polyM-specific activity, such as AlgA from *Pseudomonas* sp. E03 [34], ALYII from *Pseudomonas* sp. OS-ALG-9 [32], the AlyA from *Azotobacter chroococcum* 4A1M [31], AlgL from *Pseudomonas aeruginosa* [30], AlyA from *Pseudomonas aeruginosa* [36], AlyA from *Pseudomonas* sp. strain KS-408, and AlyM from unknown marine bacterium [33,35]. They all displayed preference to polyM substrate and very low activity toward polyG substrate. However, compared with these characterized enzymes, AlgNJ-07 showed no activity toward polyG, indicating it is a novel alginate lyase with strict polyM-specific substrate specificity.

The kinetics of AlgNJ-07 towards sodium alginate and polyM were calculated according to the hyperbolic regression analysis. As shown in Table 2, the *K_m_* values of AlgNJ-07 with sodium alginate and polyM as substrates were 0.53 mM and 0.27 mM. The results showed that AlgNJ-07 had a much lower *K_m_* values towards polyM than sodium alginate, indicating that it showed higher affinity towards polyM than that to sodium alginate. The *k_cat_*/*K_m_* values of AlgNJ-07 towards polyM (115 mM^−1^·s^−1^) was higher than alginate (64 mM^−1^·s^−1^), which indicates that the enzyme possesses higher catalytic efficiency towards M block than to MG block. The polyM-specific alginate lyase AlgL from *Pseudomonas aeruginosa* showed different *K_m_* and *k_cat_* values towards polyM substrates with various Dps and it exhibited different affinity and catalytic efficiency towards those substrates. The variation in *k_cat_*/*K_m_* with substrate length suggests that AlgL operates in a processive manner [30].

### 2.4. Biochemical Characterization of AlgNJ-07

**Figure 3:**
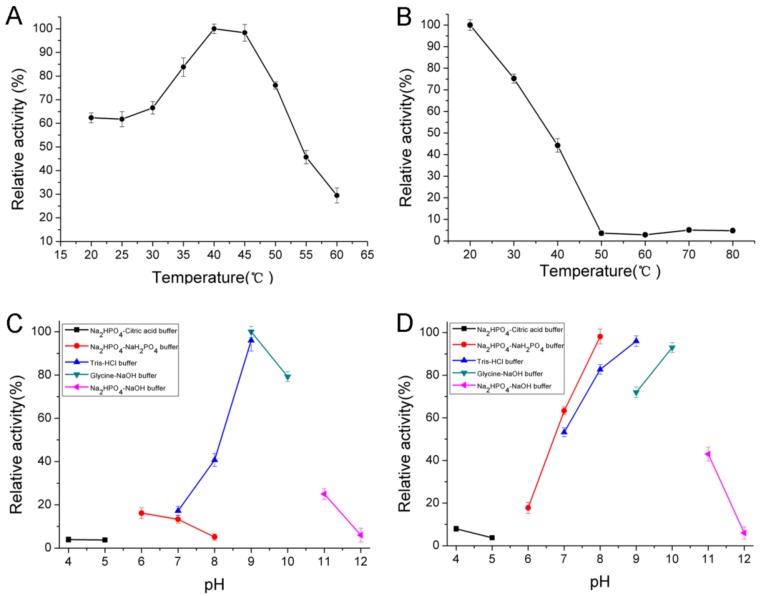
The biochemical characteristics of AlgNJ-07. (**A**) The optimal temperature of AlgNJ-07. (**B**) The thermal stability of AlgNJ-07. (**C**) The optimal pH of AlgNJ-07. (**D**) The pH stability of AlgNJ-07. Each value represents the mean of three replicates ± standard deviation.

**Figure 4:**
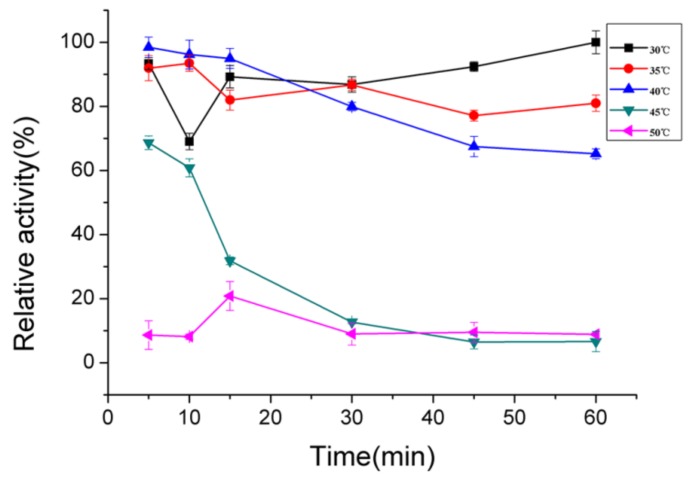
The thermal degeneration curve of AlgNJ-07. The maximal activity of the treated enzyme was regarded as 100% and the other relative activity was determined.

The enzyme showed maximum activity at 40 °C (Figure 3A) and was stable below 40 °C (Figure 3B). It possessed approximately 50% activity after incubation at 40 °C for 30 min and was gradually inactivated as the temperature increased. The thermal degeneration curve of AlgNJ-07 was shown in Figure 4. The enzyme could retain more than 70% of its total activity after being incubated at 40 °C for 60 min, which indicates it possesses better thermal stability. The optimal temperature for polyM-specific alginate lyase from *Pseudomonas* sp. strain KS-408 was 37 °C [33]. The AlgA from *Pseudomonas* sp. E03 and ALYII from *Pseudomonas* sp. OS-ALG-9 both exhibited their maximal activity at 30 °C [33,34]. While the AlyA from *Azotobacter chroococcum* 4A1M showed the highest activity at 60 °C, which shows potential in industrial applications [31].

The optimal pH for the enzyme activity was 9.0 (Figure 3C) and retained more than 80% activity at a broad pH range from pH 8.0 to 10.0 (Figure 3D) after incubation for 24 h. However, this enzyme was mostly stable at pH 9.0 and retained more than 80% activity at a broad pH range from 7.0 to 10.0. Interestingly, it could retain about 40% of its activity at pH 11.0. Thus, AlgNJ-07 was an alkaline-stable lyase and it could retain stability in a broader pH range. While most of the other characterized polyM-specific alginate lyases exhibited their maximal activity around neutral pH. For instance, the AlgA from *Pseudomonas* sp. E03 possessed its optimal pH of 8.0 [34], the AlyA from *Pseudomonas* sp. strain KS-408 displayed its maximal activity at pH of 9.0 [34]. While the AlyA from *Azotobacter chroococcum* 4A1M had a lower optimal pH of 6.0 [31].

The effects of metal ions on the activity of AlgNJ-07 are shown in Table 3. It was observed that Na^+^ could enhance the activity of the enzyme, while some divalent ions such as Zn^2+^, Cu^2+^, Mn^2+^, and Co^2+^ inhibited the activity. Interestingly, the reported activators such as Mg^2+^ and Ca^2+^ displayed slight inhibitory effects on activity of AlgNJ-07. While Ca^2+^ can activate the activities of the AlyA from *Pseudomonas* sp. strain KS-408 [33], the AlyA from *Pseudomonas* sp. E03 [34], the AlyA from *Azotobacter chroococcum* 4A1M [31], and ALYII from *Pseudomonas* sp. OS-ALG-9 [32] could enhance the substrate-binding ability of the enzyme.

**Figure 5:**
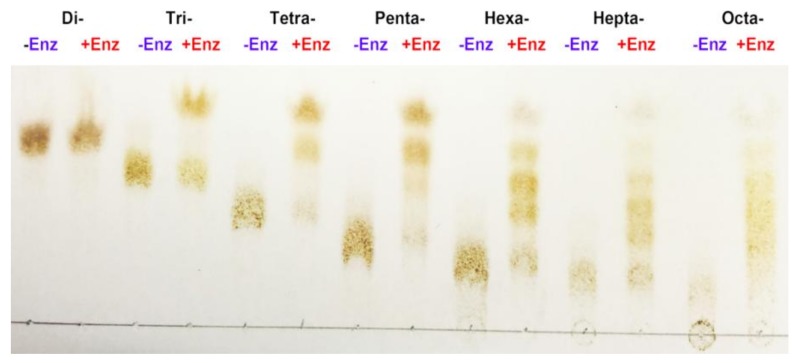
TLC analysis of hydrolysis products of oligosaccharides with Dps (2–8) for determination of substrate binding sites of AlgNJ-07 (−Enz: enzyme free; +Enz: AlgNJ-07 added).

To determine the number of substrate binding subsites in the active tunnel of AlgNJ-07, we compared the degrading capability of AlgNJ-07 to oligosaccharide substrates with different Dps. As shown in Figure 5, purified disaccharide cannot be further degraded by the enzyme even under more focused conditions (high enzyme concentration and prolonged incubation time). The trisaccharide was the shortest chain that can be recognized and cleaved by AlgNJ-07, producing monosaccharide and disaccharide. The result indicated that trisaccharide was the shortest substrate for AlgNJ-07.

**Figure 6:**
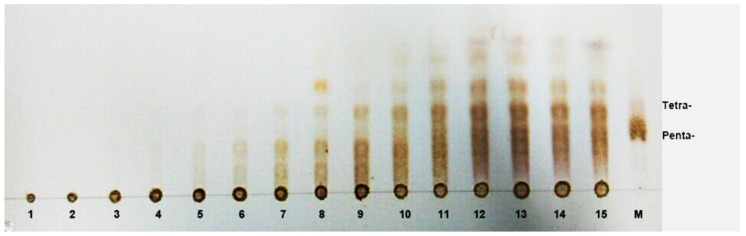
TLC analysis of the AlgNJ-07 hydrolysis products for different times. Lane 1–15, the samples taken by 0 min, 1 min, 3 min, 5 min, 10 min, 15 min, 30 min, 45 min, 60 min, 2 h, 4 h, 12 h, 24 h, 36 h, and 48 h. Lane M, the oligosaccharide standards of tetramer and pentamer.

The degradation products of sodium alginate and polyM by AlgNJ-07 were analyzed by TLC plate (Figure 6). As the proceeding of hydrolysis, oligosaccharides with high Dp (6–8) appeared. After incubation for 48 h, dimers, trimers, and tetramers turned out to be the main hydrolysis products for sodium alginate and polyM. Interestingly, the enzyme could release monosaccharide with processing of the hydrolysis. The distributions of the degradation products for the above two kinds of substrates were similar, and the results indicate that AlgNJ-07 can hydrolyze the substrates in a unique endolytic manner.

**Figure 7:**
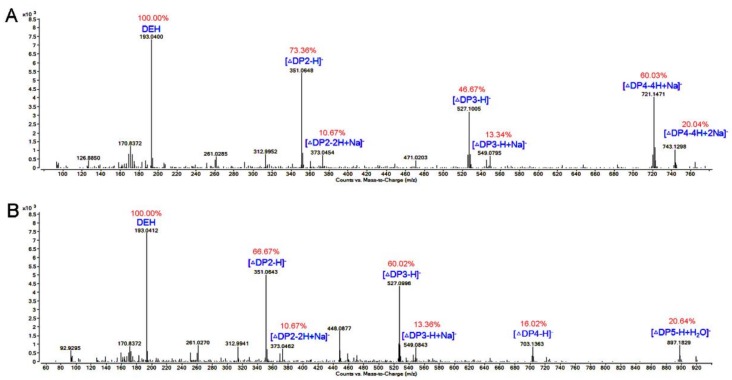
ESI-MS analysis of the degradation products of AlgNJ-07 with (**A**) alginate and (**B**) the polyM as substrate. The data highlighted in red represent the relative abundance of peaks.

In order to further determine the composition of the degradation products, the hydrolysates (1 mL) were then loaded onto a carbograph column to remove salts after removing other proteins, followed by being concentrated, dried, and re-dissolved in 1 mL methanol with the final concentration of 1 mg/mL. The degradation products were then analyzed by ESI-MS. As shown in Figure 7, monosaccharides, disaccharides, and trisaccharides account for a major fraction of the hydrolysates of two kinds of substrates. This result indicate that AlgNJ-07 may be a potential tool for the enzymatic hydrolysis of sodium alginate to produce oligosaccharides with lower Dps. The distribution of degradation products of other polyM-specific enzymes is similar, such as AlgA from *Pseudomonas* sp. E03 [34] and AlyA from *Pseudomonas* sp. strain KS-408 [33], which mainly produced oligosaccharides with Dp of 2–5 in an endolytic manner. However, the AlgL from *Pseudomonas aeruginosa* generated dimeric and trimeric products, and the rapid-mixing chemical quench studies indicate that AlgL can operate as an exopolysaccharide lyase [30]. None of those enzymes could produce monosaccharide during the hydrolytic procedure, which indicates that the AlgNJ-07 possesses a unique manner for releasing products.

## 3. Materials and Methods

### 3.1. Materials

Sodium alginate derived from brown seaweed was purchased from Sigma (St. Louis, MO, USA). PolyM (purity: about 99%) and polyG (purity: about 99%) were purchased from Qingdao BZ Oligo Biotech Co., Ltd. (Qingdao, China). The SOURCETM 15Q 4.6/100 PE column was purchased from GE HealthCare Bio-Sciences (Uppsala, Sweden). Other chemicals and reagents used in this study were of analytical grade.

### 3.2. Screening and Identification of Strain NJ-07

The samples were collected from the coast of the Yellow Sea, washed by sterilized sea water and then spread on sodium alginate-agar plates. The plates were incubated at 30 °C for 36 h and the positive colonies showing clear zones were picked out from the selection plates. The re-screening process was conducted as follows. Strains with clear hydrolytic zones were selected and incubated aerobically in a fermentation medium (modified marine broth 2216 medium containing 5 g/L (NH_4_)_2_SO_4_, 19.45 g/L NaCl, 12.6 g/L MgCl_2_·6H_2_O, 6.64 g/L MgSO_4_·7H_2_O, 0.55 g/L KCl, 0.16 g/L NaHCO_3_, 1 g/L ferric citrate, and 10 g/L sodium alginate) at 30 °C and 200 rpm. Furthermore, the activity of alginate lyase was determined by 3,5-dinitrosalicylic acid (DNS) colorimetry [37]. Among the isolates, the most active strain NJ-07 was selected for further studies. To identify the NJ-07 strain, the 16S rRNA gene of the strain was amplified through PCR by using universal primers. The purified PCR fragment was sequenced and compared with reported 16S rRNA sequences in GenBank by using BLAST. A phylogenetic tree was constructed using CLUSTAL X and MEGA 6.0 through neighbor-joining method [38].

### 3.3. Production and Purification of the Alginate Lyase

The strain NJ-07 was propagated in a fermentation medium with shaking for 40 h at 30 °C. The culture medium was centrifuged (10,000× *g*, 60 min) to completely remove the sludge and the cell-free supernatant was fractionated at 30% and 80% ammonium sulfate saturation. The precipitated protein with 30% ammonium sulfate saturation was discarded, and the precipitated protein with 80% ammonium sulfate saturation was suspended in distilled water and dialyzed in a dialysis bag (MWCO: 8000–14,000 Da) against the distilled water and freeze-dried successively. Protein contents were determined by the Bradford method [39]. The obtained enzyme powder was dissolved in 5 mL Tris-HCl buffer (pH 9.0) with 4% as the final concentration, then the enzyme solution was applied to a SOURCETM 15Q 4.6/100 PE column equilibrated with a linear gradient of 0–0.5 M NaCl in an equilibrating buffer under a flow rate of 1 mL/min. The eluents were monitored continuously at 280 nm for protein and fractions were assayed for activity against sodium alginate. Fractions were collected and monitored for the presence of alginate lyase. The purity of the fractions was assessed by SDS-PAGE. Pure fractions with activity were stored at −80 °C.

### 3.4. Enzyme Activity Assay

The purified enzyme (0.1 mL) was mixed with 0.9 mL Tris-HCl (20 mM, pH 8.0, 1% sodium alginate) and incubated at 40 °C for 10 min. The reaction was stopped by heating in boiling water for 10 min. The enzyme activity was then assayed by measuring the increased absorbance at 235 nm due to the formation of double bonds between C4 and C5 at the nonreducing terminus by β-elimination. One unit was defined as the amount of enzyme required to increase the absorbance at 235 nm by 0.01 per min [40].

### 3.5. Substrate Specificity and Kinetic Measurement of Alginate Lyase

The purified enzyme was reacted with 1% of sodium alginate, polyM, polyG, pectin, xylan, and heparin. The assays of enzyme activity for sodium alginate, polyM, and polyG were defined as described previously, whereas the assays for pectin, xylan, and heparin were determined by using the DNS method. The kinetic parameters of the purified enzyme toward sodium alginate and polyM were determined by measuring the enzyme activity with substrates at different concentrations (0.1–8.0 mg/mL). As sodium alginate is a polymer consisting of random combinations of mannuronic acid and guluronic acid residues. Since they both have the same molecular weight (MW), substrate molarity was calculated using the MW of 176 g/mol for each monomer of uronic acid in the polymer. The concentrations of the product were determined by monitoring the increase in absorbance at 235 nm using the extinction coefficient of 6150 M^−1^ cm^−1^. Velocity (V) at the tested substrate concentration was calculated as follows: V (mol/s) =  (milliAU/min × min/60 s × AU/1000 milliAU × 1 cm)/(6150 M^−1^ cm^−1^) × (2 × 10^−4^ L). The *K_m_* and *V_max_* values were calculated by hyperbolic regression analysis as described previously [41]. Additionally, the turnover number (*k_cat_*) of the enzyme was calculated by the ration of *V_max_* versus enzyme concentration ([E]).

### 3.6. Biochemical Characterization of AlgNJ-07

The effects of pH on the enzyme activity were evaluated by incubating the purified enzyme in buffers with different pHs (4.0–12.0) at 40 °C under the assay conditions described previously. The pH stability depended on the residual activity after the enzyme was incubated in buffers with different pH (4.0–12.0) for 24 h and then residual activity was determined at 40 °C under the assay conditions. Meanwhile, the effects of temperatures (20–60 °C) on the purified enzyme were investigated at pH 9.0. The thermal stability of the enzyme was determined at pH 9.0 under the assay conditions described previously after incubating the purified enzyme at 30–50 °C for 30 min. The buffers with different pHs used were phosphate-citrate (pH 4.0–5.0), NaH_2_PO_4_-Na_2_HPO_4_ (pH 6.0–8.0), Tris–HCl (pH 7.0–9.0), glycine-NaOH (pH 9.0–10.0), and Na_2_HPO_4_–NaOH (pH 11.0–12.0). In addition, the thermally-induced denaturation was also investigated by incubating the enzyme at 30–50 °C for 0–60 min.

The influence of metal ions on the activity of the enzyme was performed by incubating the purified enzyme at 4 °C for 24 h in the presence of various metal compounds at a concentration of 1 mM. Then, the activity was measured under standard test conditions. The reaction mixture without any metal ions was used as a control.

### 3.7. Substrate Binding Subsites of AlgNJ-07

To determine the smallest substrate and the number of substrate binding subsites in its catalytic tunnel of AlgNJ-07, hydrolysis reactions were carried out using oligosaccharides with different Dps (Dp 2–8) at a concentration of 10 mg/mL in 10 µL reaction mixture (pH 9.0). The reaction mixtures were incubated at 40 °C with AlgNJ-07 for 24 h. The hydrolysates were loaded onto a carbograph column (Alltech, Grace Davison Discovery Sciences, Carnforth, UK) to remove salts after removing proteins, and then concentrated, dried, and re-dissolved in 1 mL methanol. The degradation products were analyzed by TLC with the solvent system (1-butanol/formic acid/water 4:6:1) and visualized by heating TLC plate at 130 °C for 5 min after spraying with 10% (*v*/*v*) sulfuric acid in ethanol.

### 3.8. TLC and ESI-MS Analysis of the Degradation Products of AlgNJ-07

To investigate the degradation pattern of AlgNJ-07, the reaction mixtures (800 μL) containing 1 μg purified enzyme and 2 mg substrates (sodium alginate and polyM) were incubated at 30 °C for 0–48 h. The hydrolysis products were analyzed by TLC as above. To further determine the composition of the products, ESI-MS was used. The supernatants (2 µL) were loop-injected to an LTQ XL linear ion trap mass spectrometer (Thermo Fisher Scientific, Waltham, MA, USA) after centrifugation. Samples were introduced by direct infusion into the electrospray ionization source (ESI) and mass spectra (MS) were collected. To help elucidate the structure of the ESI-MS peaks, the MS spectra were collected concurrently by isolating specific *m*/*z* anions, and the oligosaccharides were detected in a negative-ion mode using the following settings: ion source voltage, 4.5 kV; capillary temperature, 275–300 °C; tube lens, 250 V; sheath gas, 30 arbitrary units (AU); and scanning the mass range, 150–2000 *m*/*z*.

## 4. Conclusions

An alginate lyase-producing bacterium was isolated and identified as *Serratia marcescens* NJ-07. The alginate lyase AlgNJ-07 was purified by anion-exchange chromatography. It had a molecular weight of approximately 25 kDa and exhibited the maximal activity of 2742.52 U/mg under 40 °C at pH 9.0. Additionally, AlgNJ-07 could retain more than 95% of its activity at pH range of 8.0–10.0, which indicates it possesses excellent pH-stability. It showed high activity and affinity toward polyM block and no activity on polyG block, suggesting it is a strict polyM-specific alginate lyase. TLC and ESI-MS analysis indicated that it can hydrolyze substrates in a unique endolytic manner and produce oligosaccharides with Dp of 2–5 and a large fraction of monosaccharides. Therefore, it may be a potent tool to produce alginate oligosaccharides with lower Dps.

## Figures and Tables

**Table 1:** The substrate specificity of AlgNJ-07 towards various substrates.

Substrate	Activity (U/mg)
Sodium alginate	2742.5
PolyM	3842.3
PolyG	N.D. *
Pullulan	N.D.
Pectin	N.D.
Xylan	N.D.
Heparin	N.D.

* No activity detected.

**Table 2:** The kinetics parameters of AlgNJ-07.

Substrate	Sodium Alginate	polyM
*K_m_* (mM)	0.53	0.27
*V_max_* (nmol/s)	74	67
*k_cat_* (s^−1^)	34	31
*k_cat_/K_m_* (s^−1^/mM)	64	115

**Table 3:** The effect of metal ions on activity of AlgNJ-07.

Reagent	Relative Activity (%)
Control	100 ± 0.5
K^+^ (100 mM)	87 ± 0.5
K^+^ (300 mM)	94 ± 0.3
K^+^ (500 mM)	92 ± 2.2
Na^+^ (100 mM)	106 ± 0.6
Na^+^ (300 mM)	120 ± 1.1
Na^+^ (500 mM)	103 ± 2.6
Zn^2+^	1 ± 0.3
Cu^2+^	5 ± 0.5
Mn^2+^	4 ± 0.1
Co^2+^	25 ± 0.3
Ca^2+^	90 ± 0.3
Fe^3+^	18 ± 0.1
Mg^2+^	98 ± 0.5
Ni^2+^	72 ± 1.2
